# Mutant Tau (P301L) Enhances Global Protein Translation in Differentiated SH-SY5Y Cells by Upregulating mTOR Signalling

**DOI:** 10.3390/ijms27010455

**Published:** 2026-01-01

**Authors:** Giovanni Luca Cipriano, Alessia Floramo, Veronica Argento, Salvatore Oddo, Osvaldo Artimagnella

**Affiliations:** 1 IRCCS Centro Neurolesi “Bonino-Pulejo”, Via Provinciale Palermo, Contrada Casazza, 98124 Messina, Italy; giovanniluca.cipriano@irccsme.it (G.L.C.); alessia.floramo@irccsme.it (A.F.); veronica.argento@irccsme.it (V.A.); 2 Department of Chemical, Biological, Pharmaceutical and Environmental Sciences (ChiBioFarAm), University of Messina, Viale F. Stagno d’Alcontres 31, 98166 Messina, Italy; salvatore.oddo@unime.it

**Keywords:** Tau, protein translation, mTOR pathway, neurons, SH-SY5Y

## Abstract

Altered protein synthesis plays a key role in ageing and multiple neurodegenerative diseases. In Alzheimer’s disease and other tauopathies, the intracellular accumulation of hyperphosphorylated Tau disrupts several cellular processes, including mRNA translation. Although Tau interacts with ribosomal proteins and modulates translational selectivity, its effects on global protein synthesis remain poorly understood. Studies report reduced translation in later disease stages but increased translation early in pathology. To clarify Tau’s impact in human neurons, we used SH-SY5Y cells overexpressing the P301L mutant form of Tau and quantified global protein synthesis using the SUnSET (Surface Sensing of Translation) puromycin-incorporation assay. We found that Tau-P301L expression greatly increased global translation by upregulating mTOR/S6 pathway. These effects were abolished by rapamycin treatment, indicating that Tau-driven translational upregulation is mTOR-dependent. Given that impaired translational control can disrupt synaptic plasticity and memory, Tau-induced alterations in protein synthesis may contribute to tauopathy progression and identify mTOR signalling as a potential therapeutic target.

## 1. Introduction

Tauopathies are a group of neurodegenerative disorders (e.g., Alzheimer’s disease, frontotemporal dementia, progressive supranuclear palsy) characterised by the intracellular accumulation of hyperphosphorylated Tau, cognitive impairments, and neuronal loss [1,2]. Tau is a microtubule-associated protein primarily found in neurons, where it stabilises microtubules, supports axonal transport, and maintains neuronal structure and polarity [3]. Tau also participates in synaptic plasticity, cell signalling, and genomic stability [4]. Under normal conditions, Tau is highly soluble and dynamically phosphorylated, which is essential for its physiological functions [5]. In tauopathies, Tau undergoes abnormal hyperphosphorylation, misfolding, and aggregation into insoluble neurofibrillary tangles [6]. Among Tau mutations, the P301L one causes a proline-to-leucine substitution that decreases Tau’s affinity for microtubules, making it more prone to phosphorylation and aggregation [7,8]. This pathological transformation disrupts microtubule stability, impairs axonal transport, and leads to synaptic dysfunction and neuronal death [8,9,10].

Alterations in the rate and fidelity of messenger RNA (mRNA) translation have been linked to ageing and various neurodegenerative diseases [11]. In this regard, beyond its canonical microtubule-associated role, a growing body of evidence points to a non-canonical role for Tau in the regulation of protein synthesis. Recent studies have revealed that mutant Tau directly interacts with ribosomal proteins, such as ribosomal protein S6, thereby compromising ribosomal function and selectively reshaping the cellular translational landscape. This interaction is profoundly detrimental, preventing mTOR (mechanistic/mammalian target of rapamycin)/S6 activity and reducing the translation of transcripts under their control [12,13]. Overall, this results in a significant decrease in global protein synthesis in neurons that bear a high burden of pathological Tau. Moreover, in mouse models of frontotemporal dementia (FTD), mutant Tau induces a strong reduction in the synthesis of ribosomal proteins, globally impairing the translation process [14,15]. Notably, in vitro HEK cell models and human Alzheimer’s disease brain tissue consistently show a decrease in protein synthesis and reduced proteome levels [12].

In addition to Tau/mTOR/S6 axis, mTOR activation increases Tau phosphorylation, accumulation, and aggregation in animal models and human brain tissue [16,17]. mTOR orchestrates protein synthesis via mTORC1 and mTORC2. mTORC1 is a key regulator of protein translation. It integrates nutrient, energy, growth factor, and stress signals to phosphorylate critical effectors, including 4E-BP1 and S6K1, thereby coordinating global and selective mRNA translation according to cellular conditions. Precise regulation of this signalling pathway is essential for normal physiology, and its dysregulation contributes to a wide range of diseases [18,19]. Moreover, because mTOR inhibits autophagy, hyperactive signalling reduces Tau clearance, whereas inhibiting mTOR (e.g., with rapamycin) enhances autophagy and diminishes Tau pathology in cellular and animal models [16,20,21,22,23]. In addition, mTOR influences Tau via downstream kinases (e.g., p70S6K, GSK3β) and phosphatases (e.g., PP2A) and by regulating Tau mRNA translation [24]. Conversely, Tau pathology can dysregulate mTOR signalling, creating a feed-forward loop that exacerbates neurodegeneration [25].

However, reports regarding Tau’s impact on global mRNA translation are contradictory, limiting our understanding of the underlying mechanisms. Although numerous reports indicate a general suppression of protein synthesis, other studies have observed enhanced global translation. Pathogenic Tau temporarily elevates global translation and selectively reprograms the translatome in a Drosophila model [26], and at early disease stages in the rTg4510 mouse model [27]. To resolve these discrepancies and gain mechanistic insight, it is crucial to investigate how mutant Tau influences protein synthesis in a well-controlled human neuronal in vitro model, as such an approach will enable the identification of early molecular alterations that may contribute to the onset and progression of neurodegeneration.

Here, we investigated the impact of the P301L mutant Tau protein on the whole nascent proteome of human neuroblastoma SH-SY5Y cells, one of the most widely used in vitro human neuronal models [28]. By examining the molecular mechanisms that drive Tau-dependent changes in protein synthesis, we seek to clarify the basis of translational dysregulation in tauopathy and ageing-related neurodegeneration.

## 2. Results

### 2.1. Mutant Tau (P301L) Affects the Nascent Proteome in Both Differentiated and Proliferative SH-SY5Y Cells

To investigate the effects of mutant Tau (P301L) on translational processes, we employed a neuroblastoma SH-SY5Y cell line, stablytransfected with mutant human tau harbouring the P301L mutation (SH-Tau) [29], and the wild-type cell line as a control (SH-wt). To quantify the impact of Tau (P301L) on protein synthesis, we took advantage of the non-radioactive SUnSET (Surface Sensing of Translation) method [30]. This is based on puromycin incorporation into newly synthesised peptides, as a structural analogue of aminoacyl-transfer RNA (tRNA). Puromycin stops translation by prematurely releasing the polypeptide chain by mimicking the 3′ end of a tRNA and attaching itself to the ribosome’s A site [31]. The amount of incorporated puromycin is directly proportional to the rate of protein synthesis and can be detected by Western blotting without the need for radioactive isotopes.

**Figure 1:**
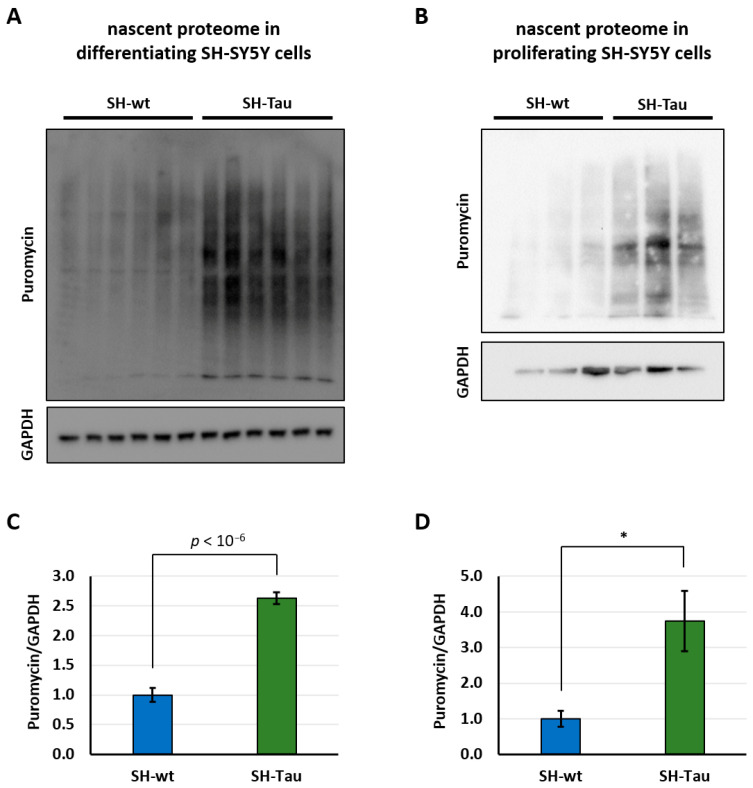
Tau (P301L) enhances rates of global protein synthesis. The rate of protein synthesis was measured via the SUnSET protocol. This evaluation was performed in both RA-differentiated (**A**) and proliferative SH-SY5Y cells (**B**), comparing SH-Tau with SH-wt. GAPDH was quantified to normalise samples. Puromycin/GAPDH levels were graphed for respective cell culture types, normalising against controls (SH-wt) (**C**,**D**). Statistical evaluation of results was performed by *t*-test, two-tailed, unpaired, and homoscedastic. * *p* < 0.05. Six biological replicates were employed for differentiated SH-SY5Y cells, and three replicates for proliferative ones. Error bars indicate s.e.m.

To achieve our goal, we first analysed and compared the nascent proteome of retinoic acid (RA)-differentiated SH-Tau and SH-wt cells, as well as that of their proliferating counterparts. We performed the SUnSET protocol on day in vitro 10 (DIV10) in both differentiative and proliferative cells. Specifically, we administered 10 µg/mL puromycin to cell cultures for 30 min. Afterwards, puromycin-labelled peptides were detected by Western blot, using anti-puromycin antibody. GAPDH was used as a loading control. We found that both differentiating and proliferating SH-Tau showed strikingly higher levels of puromycin detection than SH-wt, (Figure 1A–D). These data suggest that the mutant Tau (P301L) enhances the rate of protein synthesis in an in vitro human SH-SY5Y model.

### 2.2. Mutant Tau (P301L) Protein Increases Activation of mTOR Pathway in Differentiated SH-SY5Y Neurons

**Figure 2:**
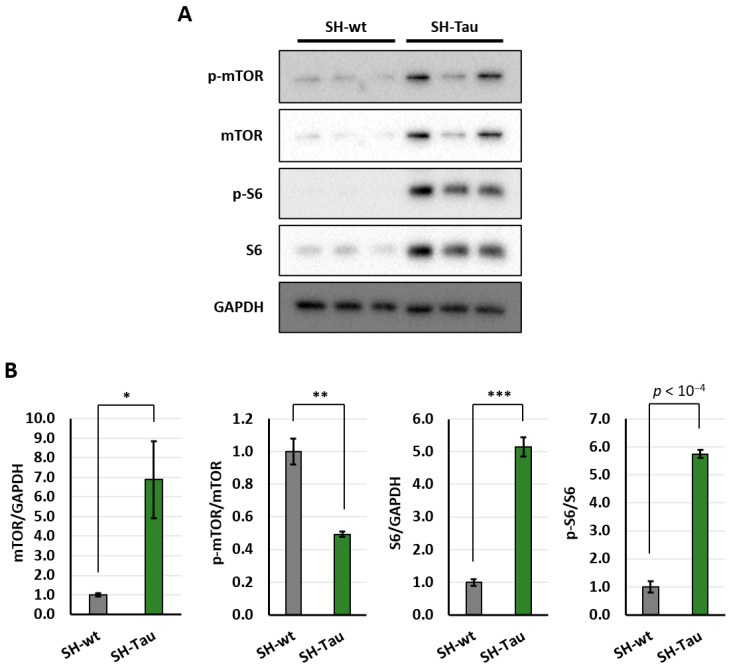
Tau (P301L) increases mTOR signalling. Western blot analyses of p-mTOR, mTOR, p-S6, and S6 proteins between differentiated SH-Tau and SH-wt cells (**A**). GAPDH was used to normalise samples relative to mTOR and S6 levels. P-mTOR and p-S6 were compared to their respective total protein levels (i.e., mTOR and S6, respectively). Protein levels were graphed, normalising against controls (SH-wt) (**B**). Statistical evaluation of results was performed by *t*-test, two-tailed, unpaired, and homoscedastic. * *p* < 0.05, ** *p* < 0.01, *** *p* < 0.001. Three biological replicates were employed for each condition. Error bars indicate s.e.m.

To explore the potential molecular mechanisms underlying the Tau-induced enhancement of the nascent proteome, we focused on differentiated neuronal cells. Recently, it has been reported that hyperphosphorylated Tau physically interacts with ribosomes, leading to a reduced global translation [12,13]. Given the role of mTOR in protein translation and the known interaction Tau/mTOR, we next analysed levels of total mTOR and S6 proteins, as well as their phosphorylated forms. Interestingly, we found that the levels of total mTOR and S6 proteins were higher in SH-Tau cells than in SH-wt cells (Figure 2). Curiously, phosphorylation of mTOR was inhibited in SH-Tau neurons, whereas the phosphorylation of S6 ribosomal protein was upregulated (Figure 2), suggesting a putative negative-feedback mechanism. Assessing mTOR activity via phosphorylation of downstream substrates is more accurate than measuring mTOR phosphorylation itself [32]. Altogether, these data demonstrate that the mTOR pathway, especially the S6 effector, is activated by mutant Tau (P301L) in differentiated SH-SY5Y neurons.

### 2.3. Mutant Tau (P301L) Upregulates Translation via mTOR Pathway in Differentiated SH-SY5Y Neurons

To confirm that mutant Tau (P301L) increases the rate of protein synthesis via activation of mTOR pathway, we measured the nascent proteome after blocking mTOR activity. For this purpose, we utilised rapamycin, a well-known inhibitor of the mTOR pathway, which ultimately blocks the phosphorylation of S6 ribosomal protein and, consequently, translation. Specifically, on DIV10, we pre-treated neurons with 20 nM rapamycin for 4 h (SH-wt rapa+ and SH-Tau rapa+) or with DMSO as a control (SH-wt rapa− and SH-Tau rapa−). Afterwards, we administered puromycin in all samples to label newly synthesised peptides.

**Figure 3:**
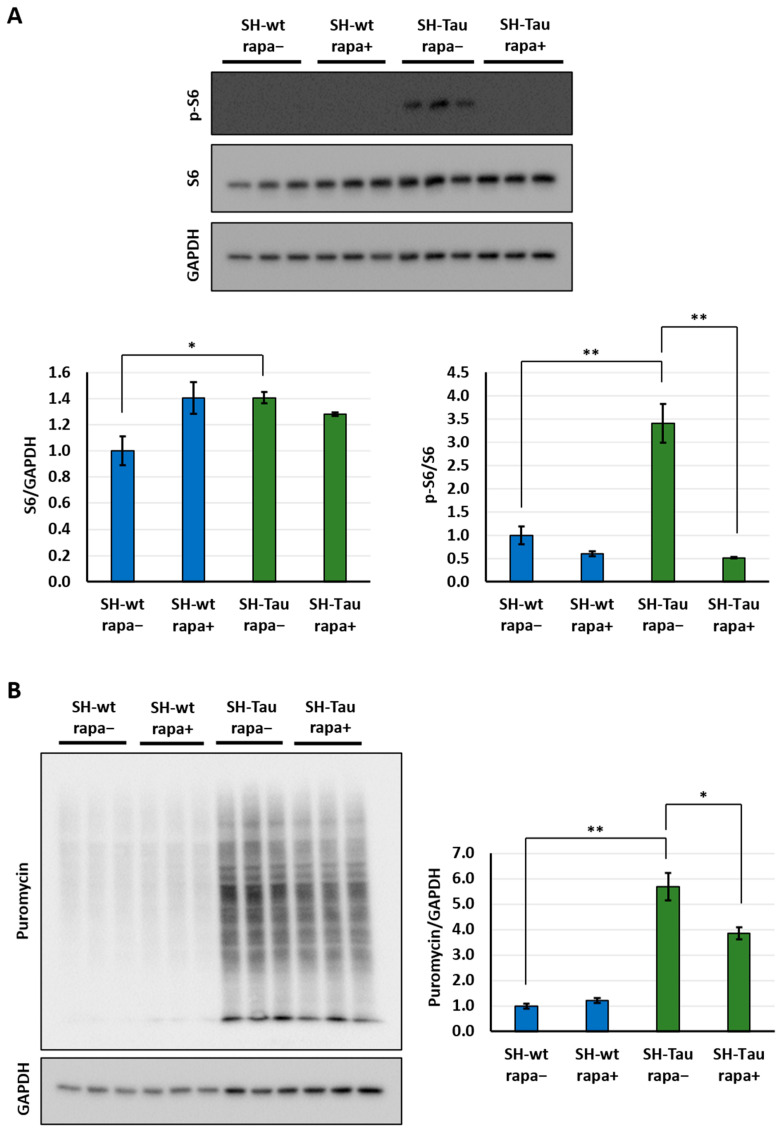
Tau (P301L) enhances nascent protein synthesis by activating mTOR signalling. S6 and p-S6 protein levels were quantified via Western blotting among four sample conditions: SH-wt rapa−, SH-wt rapa+, SH-Tau rapa−, and SH-Tau rapa+ (**A**, up). GAPDH was used to normalise samples relative to S6 levels. In contrast, p-S6 was compared to S6 protein levels and graphed (**A**, bottom). Western blot analyses of puromycin-labelled peptides under the same conditions (**B**, left). Puromycin levels were normalised against GAPDH protein and graphed (**B**, right). Throughout the figure, protein levels were graphed, normalising against controls (SH-wt rapa−). Statistical evaluation of results was performed by *t*-test, two-tailed, unpaired, and homoscedastic. * *p* < 0.05, ** *p* < 0.01. Three biological replicates were employed for each condition. Error bars indicate s.e.m.

As expected, rapamycin significantly decreased p-S6 levels in the SH-Tau condition, while the changes in p-S6 levels did not achieve statistical significance in the SH-wt condition, probably due to significantly lower baseline signal in SH-wt samples compared to SH-Tau ones. Furthermore, total S6 protein levels were comparable across samples, except for SH-Tau rapa−, which exhibited elevated levels relative to SH-wt rapa− (Figure 3A).

Next, we evaluated the functional consequences on protein translation via SunSET. As expected, we again observed upregulation of the nascent proteome in SH-Tau rapa− with respect to SH-wt rapa−. Importantly, we observed in SH-Tau neurons that rapamycin administration lowered the rate of protein synthesis, as shown by comparing SH-Tau rapa− and SH-Tau rapa+ samples (Figure 3B). The results confirmed that the mTOR pathway is essential for the upregulation of translation induced by pathological Tau protein. Notably, we did not observe a comparable reduction in protein synthesis in SH-wt samples following rapamycin administration.

Altogether, these results confirm that pathological Tau (P301L) protein regulates translation processes via the mTOR pathway in differentiated SH-SY5Y neurons. Further studies are needed to investigate how mutant Tau affects mTOR signalling.

## 3. Discussion

Altered mRNA translation is increasingly recognised as a key feature of ageing and neurodegenerative diseases, including tauopathies [11,33]. The P301L Tau mutation reduces Tau’s affinity for microtubules and enhances its phosphorylation and aggregation [34]. Beyond its canonical role in microtubule stabilisation, Tau also regulates protein synthesis. However, in vivo studies reported opposing findings, showing on one hand downregulation of translation [12,14], and on the other hand upregulation of translation [26,27]. To resolve this discrepancy, more mechanistic studies are required to unveil the molecular mechanisms of how pathological Tau modulates protein synthesis.

In this regard, our study aimed to investigate the pathological effects of mutant Tau (P301L) on translational processes in an in vitro human SH-SY5Y model. Throughout the study, we compared genetically modified cells, overexpressing mutated Tau (P301L; SH-Tau), to wild-type cells (SH-wt). Moreover, the impact on translation was assessed via the puromycin-based, non-radioactive SUnSET method, which is suitable to measure the rate of protein synthesis by Western blot [30].

Surprisingly, we found that SH-Tau showed a higher rate of translation than SH-wt; this was true in proliferating and differentiated cells. This result was unexpected because other groups reported, in different in vitro models, an inhibitory effect of pathological Tau on translation. For instance, in both inducible HEK cells and primary neurons derived from tauopathy mouse models, overall translation rates were reduced, the p-S6/S6 ratio was decreased, and ribosome complex formation was altered, as shown by Western blot analyses and polysome profiling [12,13,15]. However, findings from whole-animal studies remain inconsistent, largely depending on the labelling techniques and transgenic models used. While some evidence suggests elevated protein synthesis during early disease stages, such as in rTg4510 mice at three months old [27], other studies report decreased translation in neurons and brain regions with high pathological Tau accumulation in older mice [12]. Moreover, a recent study by Zuniga et al. [26] showed a Tau (R406W)-mediated positive effect on translation in an early disease Drosophila model, increasing levels of growth-promoting factors, postsynaptic proteins, enzymes associated with oxidative metabolism, and proteins affecting Tau proteolysis. In addition, some studies supported the idea that an increment of translation, via p-PERK/p-eIF2α inhibition, exerts neuroprotective roles at the early stage of tauopathy [27,35]. Collectively, as discussed also in Zuniga’s study, these observations suggest that protein synthesis is suppressed in neurons heavily burdened by Tau pathology, whereas global translational activity may be transiently enhanced at early stages of tauopathy, potentially reflecting an adaptive mechanism of neurons to limit neurodegeneration. In this regard, our findings in human neuroblastoma SH-SY5Y cells suggest that it may recapitulate early stages of tauopathies.

From a molecular point of view, the ribosome apparatus and mTOR signalling are crucial in tauopathy. It has been demonstrated that hyperphosphorylated Tau interacts with S6 ribosomal protein, preventing its activity [12], and associates with ribosomes as well [13]. Moreover, pathogenic Tau regulates gene expression of some key genes involved in translation, including ribosomal subunits [36], thereby impacting the overall protein synthesis process. Interestingly, growing evidence suggests a molecular interplay between Tau and mTOR [16,25,37]. Here, we report that Tau (P301L) activates mTOR pathway, especially upregulating the phosphorylation levels of the final effector, S6 ribosomal protein. Finally, we found that inhibiting mTOR activity by rapamycin reduced the Tau-induced increase in total protein translation. Taken together, these data suggest that mTOR pathway is necessary and mechanistically associated with the Tau-mediated upregulation of the nascent proteome in RA-differentiated SH-SY5Y neurons.

mTOR-dependent translational control is widely reported to be crucial in synaptic plasticity and memory. To this end, mTOR inhibition blocks long-term potentiation, synaptic facilitation, and long-term memory formation, as reviewed in [38,39]. Nevertheless, a large body of evidence shows that hyperactive mTOR signalling can adversely impact multiple forms of learning and memory [40,41]. In this view, mTOR/S6 pathway could be a potential therapeutic target to ameliorate translational impairments and, consequently cognitive disfunctions related to tauopathy progression.

### Limitations and Follow-Ups

Although our findings clearly demonstrate that Tau (P301L) enhances mTOR activity and increases protein synthesis, it is important to acknowledge the intrinsic biological differences in an in vitro model such as the SH-SY5Y cell line. As with any cellular model, complementary studies in additional systems will further strengthen the generalizability of these results. To this end, our data strongly indicate that Tau P301L elevates global translation through mTOR signalling, as supported by the robust increase in phosphorylation of the downstream effector S6. These results provide compelling evidence that mutant Tau activates the mTOR pathway and thereby regulates protein synthesis. Future validation in more physiologically relevant systems, such as induced pluripotent stem cell (iPSC)-derived neurons, would expand the translational relevance of our findings. iPSC-based models also offer the opportunity to introduce specific MAPT mutations through gene editing and to differentiate cells into well-defined neuronal subtypes.

Despite the well-established role of mTOR in regulating autophagy [42], we unexpectedly observed no significant changes in the steady-state levels of key autophagy-related proteins, such as Beclin-1 and LC3 (Figure S2). This observation would be more rigorously validated by assessing autophagic flux using dedicated functional assays. Similarly, levels of the amyloid precursor protein (APP) were not altered by expression of mutant Tau (P301L) (Figure S2). This is consistent with previous findings showing that increased Tau levels in a mouse model of Alzheimer’s disease do not alter APP steady-state levels or affect Aβ pathology [43].

Additional mechanistic work will be valuable to fully delineate how mutant Tau engages the mTOR pathway. Transcriptomic profiling and network analyses could help identify upstream regulators and downstream targets influenced by Tau P301L. Moreover, to determine which transcripts are translationally regulated, ribosome profiling represents the most informative approach. This technique allows for quantitative assessment of all ribosome-engaged mRNAs and would reveal which genes are translationally up- or downregulated by mutant Tau. Previous ribosome profiling studies in mice and Drosophila have shown that mutant Tau alters the translation of ribosomal subunits and key neurodegeneration-related factors, suggesting that similar regulatory patterns may occur in human neuronal models [12,15,26].

## 4. Materials and Methods

### 4.1. Cell Culture and Differentiation

The human neuroblastoma cell line SH-SY5Y (CRL-2266™, ATCC, Manassas, VA, USA) and the SH-SY5Y cell line overexpressing the TAU P301L mutation (P30722, Innoprot, Derio, Bizkaia, Spain) were used for the experiments. Cells were maintained as monolayers at 37 °C in a humidified atmosphere containing 5% CO_2_. Wild-type SH-SY5Y (SH-wt) cells were cultured in RPMI-1640 medium (#R0883, Sigma-Aldrich, Saint Louis, MO, USA) supplemented with 10% foetal bovine serum (FBS, #F7524, Sigma-Aldrich), 1% L-glutamine (#G7513, Sigma-Aldrich), and 1X penicillin-streptomycin (P/S, #P0781, Sigma-Aldrich). SH-SY5Y-TAU P301L (SH-Tau) cells were maintained in the same medium, replacing P/S with the addition of 300 µg/mL G418 disulfate (#J62671, Thermo Fisher Scientific, Rochester, NY, USA).

On day in vitro 0 (DIV0), both cell lines were cultured in 6-well plates (#130184, Thermo Scientific) at densities of 300,000 cells/well and 400,000 cells/well for SH-Tau and SH-wt conditions, respectively. Different seeding densities were chosen to compensate for the differing proliferation rates of the two cell lines on DIV1, the time of neuronal differentiation induction. Differentiation was induced by supplementing the medium with 10 µM retinoic acid (RA) (#R2625, Sigma-Aldrich) [44,45]. In detail, we kept cultures in differentiation medium for a total of ten days. To ensure proper differentiation, we progressively reduced the FBS concentration (2.5% from DIV1 to DIV6, and then 1%). Differentiation was considered complete by day 10 (DIV10).

Proliferative (non-differentiated) SH-SY5Y cell cultures were constantly maintained with 10% FBS from DIV0 to DIV10. In this case, cells were plated in 6-well plates at densities of 100,000 cells/well for SH-Tau and 130,000 cells/well for SH-wt cells, ensuring comparable time points with differentiated cultures.

### 4.2. Puromycin and Rapamycin Treatment

Following neuronal differentiation, on DIV10, cells were treated with 10 μg/mL puromycin (#P8833, Sigma-Aldrich) for 30 min, which was added directly to the culture medium, according to the SUnSET (Surface Sensing of Translation) protocol [30]. This method relies on the incorporation of puromycin, a structural analogue of aminoacyl-tRNA, into nascent polypeptide chains, which leads to premature termination of translation. The amount of incorporated puromycin is directly proportional to the rate of protein synthesis and can be detected using anti-puromycin antibodies. The ideal time of puromycin administration and protein quantity to load in Western blot assays were previously tested (Figure S1). After the incubation period, the medium was removed and cells were washed with 1X phosphate-buffered saline (PBS; Sigma-Aldrich, Saint Louis, MO, USA) to eliminate any residual, non-incorporated puromycin. Cells were then incubated with 500 μL of trypsin (#T4049, Sigma-Aldrich) for 5 min to facilitate detachment. Trypsin activity was neutralised by adding an equal volume (500 μL) of FBS culture medium. The resulting cell suspension was centrifuged at 300× *g* for 5 min at 4 °C. Cell pellets were stored at −80 °C or immediately used for Western blot analyses.

Rapamycin was used to inhibit mTOR signalling. On DIV10, differentiated cells were pre-treated with 20 nM rapamycin (#553210, Sigma-Aldrich) for 4 h prior to puromycin exposure [46,47]. Control cells were simply treated with PBS-diluted DMSO (at a concentration <0.1%) as a control. Samples were organised into four experimental groups based on genotype (wild-type or TAU P301L mutant) and treatment conditions (presence or absence of rapamycin): SH-wt rapa−, SH-wt rapa+, SH-Tau rapa−, and SH-Tau rapa+.

### 4.3. Western Blot Analyses

Cell pellets were processed for protein extraction using the NE-PER™ Nuclear and Cytoplasmic Extraction Reagents (#78835, Thermo Fisher Scientific), following the manufacturer’s protocol. The total protein concentration in each sample was determined using the Bradford assay (#5000006, Bio-Rad Laboratories, Hercules, CA, USA), allowing for equal loading of 30 µg of protein per lane during electrophoresis. Proteins were denatured in 1X sample buffer (#1610747), containing 2.5% 2-Mercaptoethanol (#M3148), at 95 °C and subsequently separated by 10% SDS-polyacrylamide gel electrophoresis (SDS-PAGE). A prestained protein ladder 10 to 250 kDa (#26619, Thermo Fisher Scientific) was loaded according to the manufacturer’s instructions. Following electrophoresis, proteins were transferred onto PVDF membranes (#IPVH00010, Immobilon-P PVDF, Merck Millipore division of Merck KGaA, Darmstadt, Germany) at 4 °C for 2 h, 100 Volt or overnight at 25 Volt (to allow detection of p-mTOR/mTOR proteins). To block non-specific binding, membranes were incubated for 1 h at room temperature in 1× TBS-Tween (TBST) containing 5% non-fat dry milk. After three washes in TBS-T, membranes were incubated overnight at 4 °C with the appropriate primary antibody specific to the target protein. The following primary antibodies were used:

- Anti p-mTOR, rabbit polyclonal (Ser2448), (1:1000; #2971S, Cell Signaling, Danvers, MA, USA);
- Anti-mTOR, rabbit polyclonal (1:1000; #2972, Cell Signaling, Danvers, MA, USA);
- Anti-p-S6, rabbit polyclonal (Ser240/244), (1:1000; #2215, Cell Signaling, Danvers, MA, USA);
- Anti-S6, rabbit monoclonal (1:1000; #2217, Cell Signaling, Danvers, MA, USA);
- Anti-puromycin, mouse monoclonal (1:5000, #MABE343-AF647; Sigma-Aldrich).

Following primary antibody incubation, membranes were washed and then incubated for 1 h at room temperature with the appropriate HRP-conjugated secondary antibody. Specifically, membranes were treated with either a mouse anti-rabbit IgG secondary antibody (1:2000; #sc-2357, Santa Cruz Biotechnology, Inc., Dallas, TX, USA) or a chicken anti-mouse IgG antibody (1:2000; #SA1-72021, Thermo Scientific, Waltham, MA, USA), depending on the species of the primary antibody. The HRP-conjugated anti-GAPDH, rabbit monoclonal, 14C10 (1:1000; #3683, Cell Signaling Technology, Danvers, MA, USA), was incubated at room temperature for 1 h. After further washing to remove unbound antibodies, protein bands were visualised using an enhanced chemiluminescence substrate (#WBLUF0500, Immobilon Forte Western HRP Substrate, Millipore Corporation, Burlington, MA, USA). The signal was then detected and acquired using the ChemiDoc™ MP Imaging System (Bio-Rad, Hercules, CA, USA), allowing for high-resolution documentation and analysis of the immunoreactive bands. The membranes were first blotted to detect phosphorylated protein forms, then they were stripped with Restore™ Western Blot buffer (#21059, Thermo Scientific, Meridian, Rockford, IL, USA), following the manufacturer’s protocol. Then, membranes were re-incubated with antibodies against the respective total forms on the same membrane. We repeated the same procedure for GAPDH detection. Otherwise, blots were sequentially probed as appropriate with antibodies against GAPDH and puromycin.

Bands were quantified by using ImageJ-Fiji 1.54f software (National Institute of Health, Bethesda, MD 20814, USA). Specifically for p-S6 evaluation, since density of bands resulted very low (approaching zero), to avoid abnormal representation of data, we included the background value in densitometric quantification.

### 4.4. Statistical Analysis

Data were analysed by Excel software. Full details of numerical and statistical analysis of data (including normalisation criteria, number of biological replicates; statistical tests employed for result evaluation) are provided in Table S1, in the figures and their legends. A *p*-value < 0.05 was considered statistically significant. Data are expressed as mean ± Standard Error of the Mean (s.e.m.).

## 5. Conclusions

This study investigated the effects of the P301L Tau mutation on protein synthesis in a human differentiated SH-SY5Y neuronal model, addressing conflicting findings in the literature and a specific gap regarding human in vitro neuronal models. We conclude that the mutant Tau (P301L) protein enhances, rather than suppresses, global protein translation in this system. This observed increase in the nascent proteome is mechanistically driven by the activation of the mTOR pathway, specifically resulting in the significant upregulation and phosphorylation of the downstream effector, ribosomal protein S6. This conclusion is strongly supported by our finding that inhibiting the pathway with rapamycin successfully reversed the Tau-mediated enhancement of protein synthesis. These results suggest that the SH-SY5Y model may recapitulate an early stage of tauopathy, where translational activity is transiently enhanced, rather than repressed as seen in later disease stages. While acknowledging the limitations of this cell model and the need for future validation in iPSC-derived neurons, this study provides clear evidence that the mutant Tau (P301L)-mTOR-S6 axis is a critical driver of translational dysregulation.

## Data Availability

The original contributions presented in this study are included in the article. Further inquiries can be directed to the corresponding author(s).

## Supplementary Materials

The following supporting information can be downloaded at: https://www.mdpi.com/article/10.3390/ijms27010455/s1.

## Abbreviations

The following abbreviations are used in this manuscript:

APP	amyloid precursor protein
DIV	day in vitro
FBS	foetal bovine serum
FTD	frontotemporal dementia
iPSC	induced pluripotent stem cell
mRNA	messenger RNA
mTOR	mechanistic/mammalian target of rapamycin
P/S	penicillin-streptomycin
PBS	phosphate-buffered saline
RA	retinoic acid
SDS-PAGE	Sodium dodecyl sulfate-polyacrylamide gel electrophoresis
s.e.m.	Standard error of the mean
SUnSET	Surface Sensing of Translation
TBST	Tris-buffered saline with Tween
tRNA	aminoacyl-transfer RNA
