# Supplementary figures and images for: Mutant Tau (P301L) Enhances Global Protein Translation in Differentiated SH-SY5Y Cells by Upregulating mTOR Signalling
Source: Int J Mol Sci. 2026 Jan 1;27(1):455. doi: 10.3390/ijms27010455 (PMC12785925; doi:10.3390/ijms27010455)

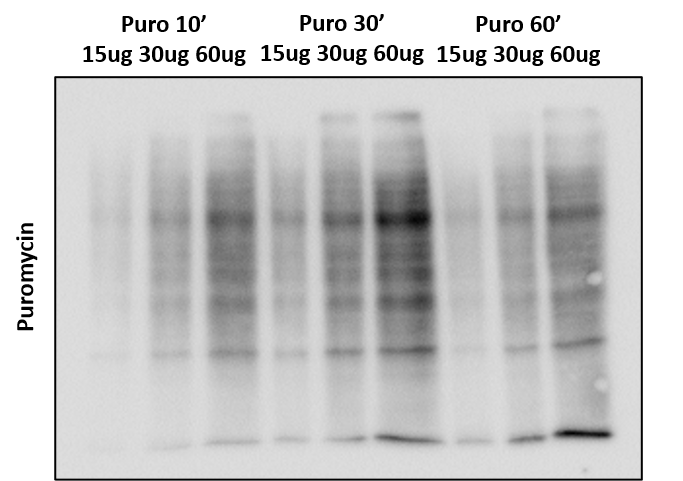

Supplement: Supplementary file 1 [file ijms-27-00455-s001.zip › Figure_S1.tif]

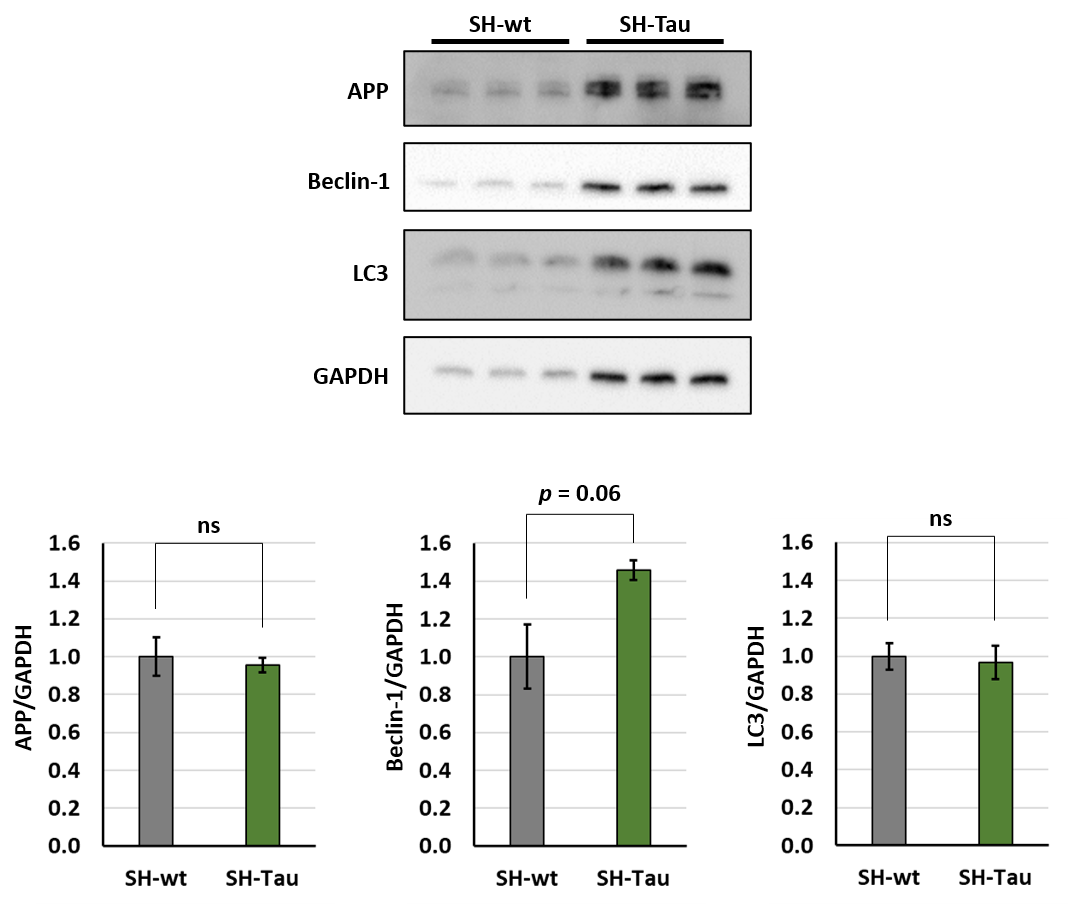

Supplement: Supplementary file 1 [file ijms-27-00455-s001.zip › Figure_S2.tif]
